# Targeting scFv-Fc-scTRAIL fusion proteins to tumor cells

**DOI:** 10.18632/oncotarget.24379

**Published:** 2018-01-31

**Authors:** Meike Hutt, Sina Fellermeier-Kopf, Oliver Seifert, Lisa C. Schmitt, Klaus Pfizenmaier, Roland E. Kontermann

**Affiliations:** ^1^ Institute of Cell Biology and Immunology, University of Stuttgart, Stuttgart 70569, Germany; ^2^ Stuttgart Research Center Systems Biology, University of Stuttgart, Stuttgart 70569, Germany

**Keywords:** TRAIL, targeting, affinity, receptor expression, apoptosis

## Abstract

Fusion proteins combining hexavalent TRAIL with antibody fragments allow for a targeted delivery and efficient apoptosis induction in tumor cells. Here, we analyzed scFv-Fc-scTRAIL molecules directed against EGFR, HER2, HER3, and EpCAM as well as an untargeted Fc-scTRAIL fusion protein for their potentials to induce cell death both *in vitro* and in a xenograft tumor model *in vivo*. The scFv-Fc-scTRAIL fusion protein directed against EGFR as well as the fusion protein directed against EpCAM showed targeting effects on the two tested colorectal carcinoma cell lines Colo205 and HCT116, while a fusion protein targeting HER3 was more effective than untargeted Fc-scTRAIL only on Colo205 cells. Interestingly, another anti-HER3 scFv-Fc-scTRAIL fusion protein exhibiting approximately 10-fold weaker antigen binding as well as the HER2-directed molecule were unable to increase cytotoxicity compared to Fc-scTRAIL. A comparison of EC_50_ values of cell death induction and antigen binding supports the assumption that high affinity antigen binding is one of the requirements for *in vitro* targeting effects. Furthermore, a minimal number of expressed target antigens might be required for increased cytotoxicity of targeted compared to non-targeted molecules. In a Colo205 s.c. xenograft tumor model, strongest antitumor activity was observed for the anti-HER3 scFv-Fc-scTRAIL fusion protein based on antibody 3-43, with complete tumor remissions after six twice-weekly injections. Surprisingly, a similar *in vivo* activity was also observed for untargeted Fc-scTRAIL in this tumor model, indicating that additional factors contribute to the potent efficacy of targeted as well as untargeted hexavalent Fc-scTRAIL fusion proteins *in vivo*.

## INTRODUCTION

Pro-apoptotic receptor agonists (PARAs) are powerful molecules to kill cancer cells independently of p53 and the intrinsic apoptosis pathway. Especially, TRAIL (tumor necrosis factor-related apoptosis-inducing ligand) that selectively induces tumor cell death without affecting healthy cells has been extensively investigated as anti-cancer therapeutic. Despite promising anti-tumor effects in preclinical models [1, 2], soluble TRAIL (sTRAIL) as well as agonistic antibodies against the two death receptors TRAIL-R1 (DR4) and TRAIL-R2 (DR5) did not show therapeutic activity in clinical trials [3]. This deficiency has been attributed to intrinsic or acquired resistance of many human tumors towards TRAIL-induced apoptosis [4–6] as well as to a limited efficacy of the applied therapeutics. This insufficient activity has meanwhile been linked to the lacking ability of sTRAIL and agonistic antibodies to induce higher order clustering of TRAIL receptors [7–9]. While soluble TRAIL is only able to activate apoptosis via TRAIL-R1 and requires multivalent display to induce apoptosis via TRAIL-R2 [10, 11], agonistic antibodies have been demonstrated to depend on secondary crosslinking via their Fc part for efficient activity [12–15]. Based on the required clustering of death receptors for efficient apoptosis induction, several strategies have been developed to enhance the activity of TRAIL-based therapeutics [8].

One approach is the fusion of TRAIL to antibody derivatives, such as single-chain variable fragments (scFv). Through binding to cell surface structures, the fusion protein is delivered to target cells and, in addition, mimics the membrane-bound form of TRAIL capable of activating TRAIL-R2. Most commonly, TRAIL has been combined with scFvs directed against tumor-associated antigens (TAA) to directly kill target-positive tumor cells in *cis* as well as target-negative cancer cells in *trans* through a bystander effect [16]. Furthermore, scFvs targeting the tumor vasculature or immune cells have been employed to either disrupt tumor supply or to equip immune cells with additional cytotoxic activity. ScFv-TRAIL fusion proteins have been shown to exert considerably higher activity compared to unmodified TRAIL due to active targeting and in some cases via activation or inhibition of signaling pathways triggered by the target antigen [7, 8, 17]. TRAIL, however, is a homotrimeric protein, which on the one hand might allow dissociation of the fusion proteins into their monomeric subunits and on the other hand limits the types of possible combinations with fusion partners. Development of single-chain variants of TRAIL (scTRAIL) comprising the extracellular part of TRAIL fused via short peptide linkers allowed the generation of a completely new set of fusion proteins with improved stability [18–21].

Another concept to improve the activity of TRAIL-based therapeutics is based on inducing death receptor oligomerization by increasing the valency of the molecules. Several studies showed that TRAIL-R2-targeting antibody fragments or scaffold proteins in an at least tetravalent format exert increased activity compared to their counterparts possessing a lower number of TRAIL receptor-binding sites [22–25]. Consistent with these data, fusion of TRAIL to an isoleucine zipper hexamerization motif [26] or dimeric assembly of scTRAIL modules considerably improved apoptosis induction [19, 20, 27].

In previous studies, we already showed that combination of tumor targeting and dimeric assembly of scTRAIL generates highly active molecules [19, 20]. Especially, fusion proteins comprising a γ1 Fc region proved to be more efficacious *in vivo* than other formats. Although targeting effects were demonstrated for all formats *in vitro* and stronger activity has been observed for EGFR-targeting scFvhu225-EHD2-scTRAIL compared to EHD2-scTRAIL *in vivo*, scFvhu225-Fc-scTRAIL and non-targeting Fc-scTRAIL showed similar potent anti-tumor effects in Colo205 xenograft models [28].

In the present study, we utilized the scFv-Fc-scTRAIL and Fc-scTRAIL formats and extended the targeting approach by employing five antibody moieties of different specificity and affinity. Besides the EGFR-targeting scFv, we incorporated antibody moieties directed against the human epidermal growth factor receptor 2 (HER2), HER3, and the epithelial cell adhesion molecule (EpCAM). We investigated the biochemical and functional properties of the different targeted and non-targeted Fc fusion proteins *in vitro* and in a Colo205 xenograft model to further study the influence of targeting on bioactivity as well as factors that determine targeting effects.

## RESULTS

### Tumor-targeted scFv-Fc-scTRAIL fusion proteins and non-targeted Fc-scTRAIL

**Figure 1:**
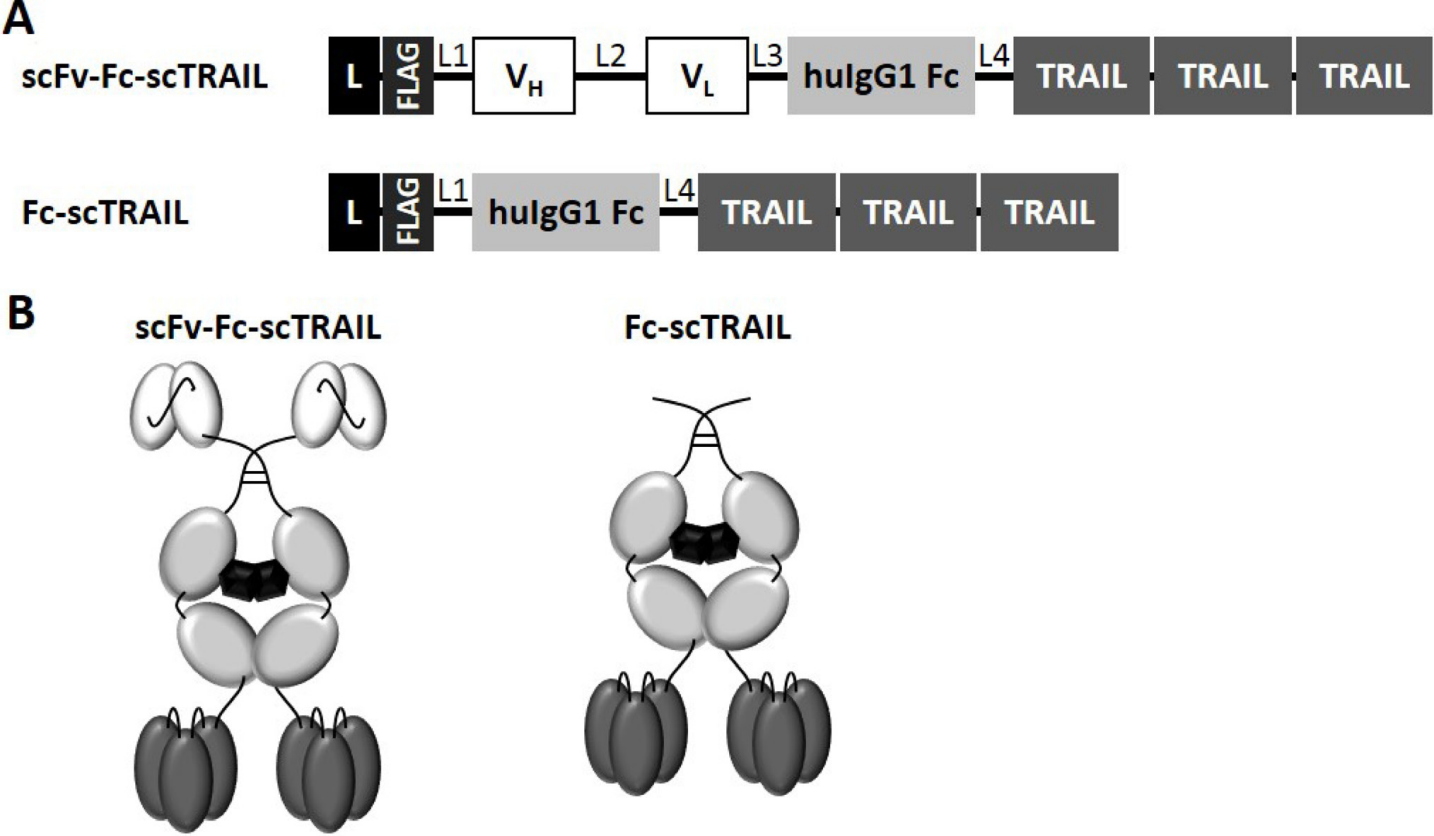
Schematic overview of scFv-Fc-scTRAIL and Fc-scTRAIL fusion proteins (**A**) Composition and (**B**) schematic assembly are shown. L, Igκ chain leader sequence. L1, GGGGSGT linker. L2, (GGGGS)_3_ linker. L3, AAAGGSGG linker. L4, GGSGGGSSGG linker. TRAIL subunits comprise aa 118-281 and are connected by a glycine residue as linker.

**Figure 2:**
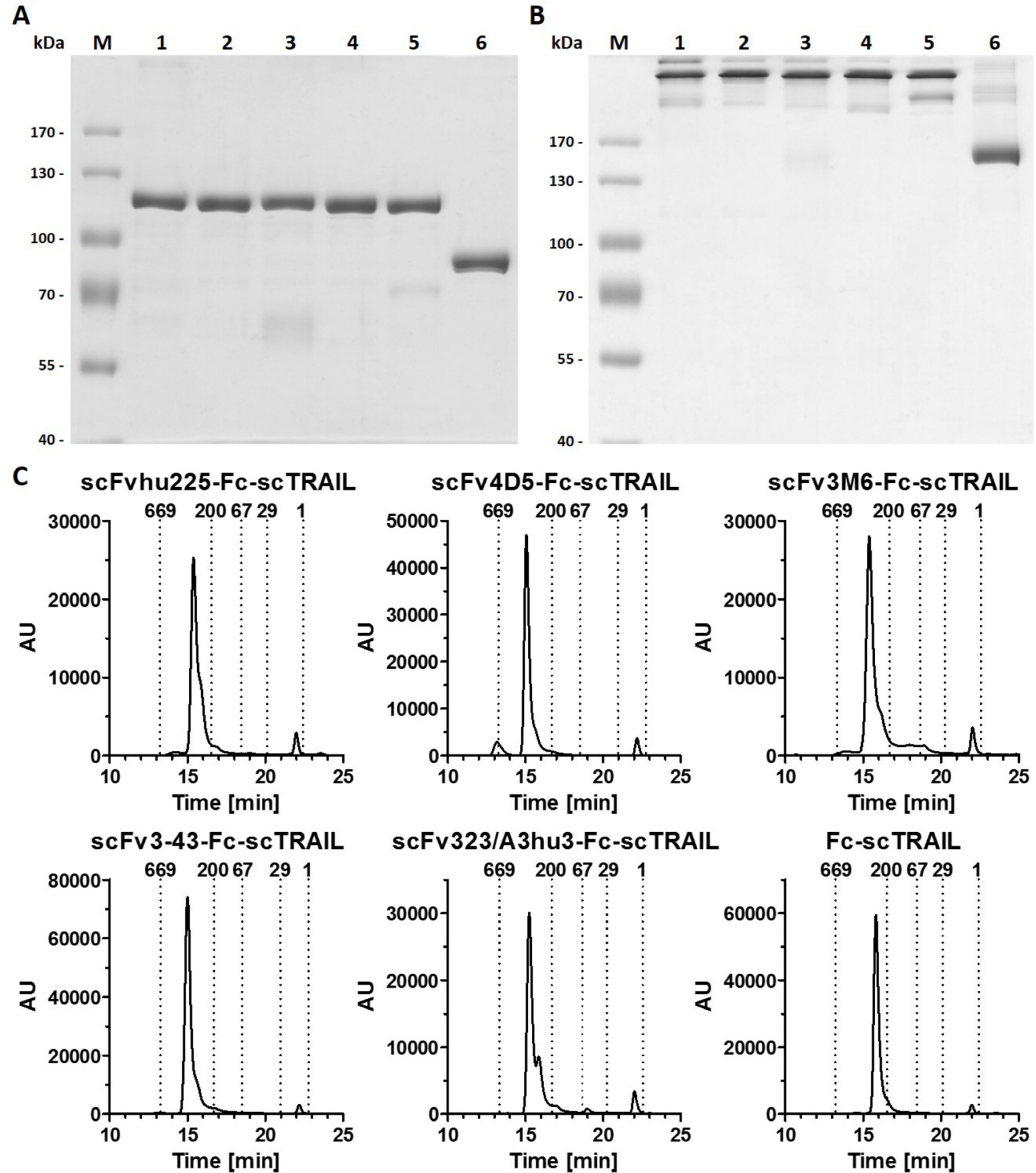
Biochemical characterization of scFv-Fc-scTRAIL and Fc-scTRAIL fusion proteins (**A**) SDS-PAGE (8% PAA) analysis of scFvhu225-Fc-scTRAIL (1), scFv4D5-Fc-scTRAIL (2), scFv3M6-Fc-scTRAIL (3), scFv3-43-Fc-scTRAIL (4), scFv323/A3hu3-Fc-scTRAIL (5), Fc-scTRAIL (6) under reducing and (**B**) non-reducing conditions. 2 µg protein were analyzed per lane (lane M, molecular mass standard). (**C**) Size exclusion HPLC of targeted and non-targeted Fc-scTRAIL molecules. Elution points of standard proteins and their respective molecular masses [kDa] are indicated.

Previously, we identified superior properties of Fc-comprising scTRAIL fusion proteins compared to other targeted and non-targeted dimeric formats [28]. Therefore, scFv-Fc-scTRAIL and Fc-scTRAIL were used in this study to investigate the influence of targeting. In these formats, a single-chain version of TRAIL that consists of amino acids 118 to 281 with a single glycine residue as linker to connect the protomers [21] was fused to the C-terminus of a human γ1 Fc region, while a TAA-targeting single-chain variable fragment was optionally located N-terminally (Figure 1A, 1B). Five different antibody moieties directed against four distinct tumor-associated antigens were employed, including the EGFR-targeting antibody hu225 derived from antibody C225 used in cetuximab [29] and humanized by CDR grafting [30], the trastuzumab-derived 4D5 directed against HER2 [31], the HER3-targeting antibodies 3M6 (a modified version of MM-121, Ab#6 [32] with a mutation of C89 of the V_L_ according to the Kabat numbering scheme to serine) and 3-43 [33], as well as the humanized version 323/A3hu3 [34] of the anti-EpCAM antibody 323/A3 [35, 36]. All molecules further comprised a FLAG-tag at the N-terminus allowing purification of the proteins from the supernatant of stably transfected HEK293T cells by FLAG affinity chromatography. Yields ranged from 3.8 to 12.7 mg protein per liter supernatant depending on the employed antibody fragment (Table 1). Purity as well as formation of disulfide-linked dimers under non-reducing conditions were confirmed by SDS-PAGE analysis (Figure 2A, 2B). In size exclusion chromatography, all proteins eluted as one major peak. However, high molecular weight species were found for scFv4D5-Fc-scTRAIL, whereas fractions of smaller size were detected for scFv3M6-Fc-scTRAIL and scFv323/A3hu3-Fc-scTRAIL (Figure 2C). Non-targeted Fc-scTRAIL exhibited a Stoke’s radius of 6.0 nm and fusion to scFv molecules increased protein size by 0.4 nm to 0.7 nm. Thermal stability was evaluated by dynamic light scattering revealing melting points of 57° C to 63° C (Table 1).

**Table 1:** Biochemical properties of scFv-Fc-scTRAIL fusion proteins and Fc-scTRAIL

Format	Target	Antibody	M_r_	Yield [mg/l]	S_r_ [nm]	T_M_ [° C]
scFv-Fc-scTRAIL	EGFR	hu225	222	10.4	6.4	58
	HER2	4D5	222	12.7	6.7	63
	HER3	3M6	221	3.8	6.6	57
	HER3	3-43	222	8.8	6.7	61
	EpCAM	323/A3hu3	223	5.2	6.7	60
Fc-scTRAIL	-	-	169	6.9	6.0	58

### Binding to tumor-associated antigens and TRAIL receptors

**Figure 3:**
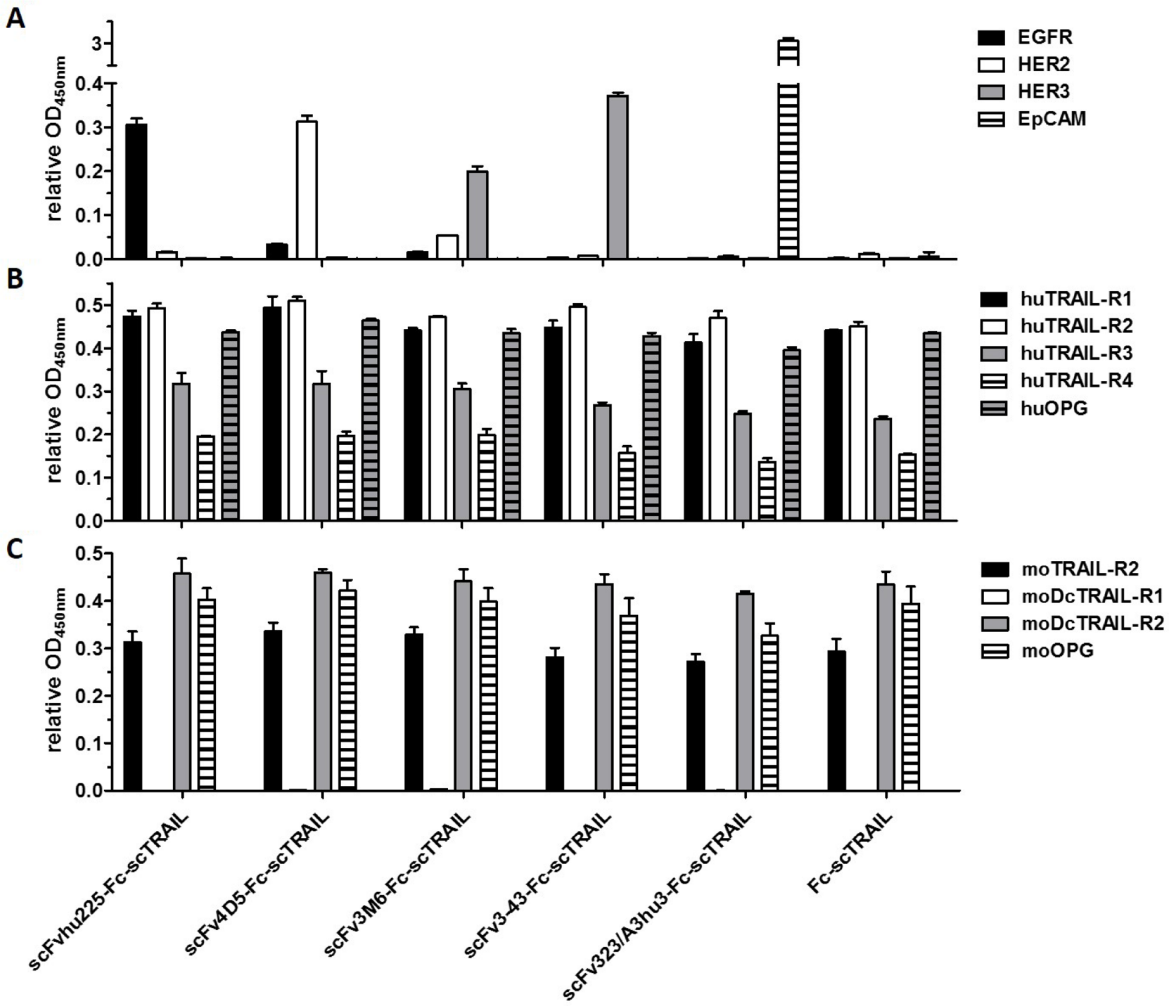
Binding to target proteins in ELISA (**A**) 20 nM protein (scTRAIL units) were analyzed for binding to receptor-Fc fusion proteins or sEpCAM (300 ng/well). Bound molecules were detected with an anti-FLAG-HRP or anti-human IgG (Fc specific)-POD. (**B**) Binding to Fc fusion proteins of human and (**C**) mouse TRAIL receptors (200 ng/well) was investigated at a concentration of 40 nM scTRAIL units. Bound molecules were detected with an anti-FLAG-HRP. Relative OD_450_ nm, OD of sample measured at 450 nm divided by OD of coating control (detected via anti-human IgG (Fc specific)-POD or anti-FLAG-HRP). Block shift was performed for binding to human TRAIL receptors.

**Figure 4:**
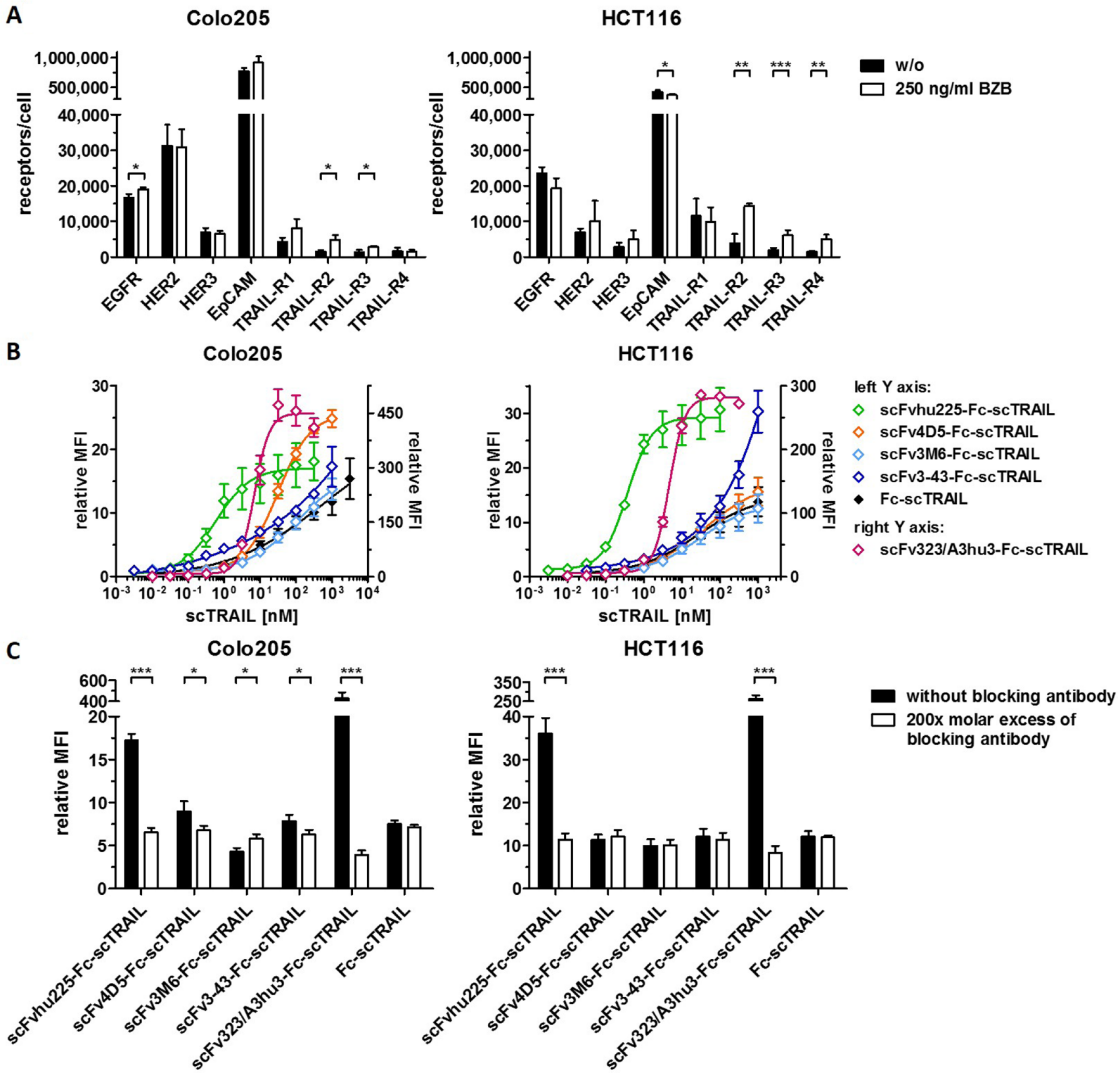
Flow cytometry studies of scFv-Fc-scTRAIL molecules and Fc-scTRAIL on Colo205 and HCT116 cells (**A**) Expression levels of EGFR, HER2, HER3, EpCAM, and TRAIL-receptors were investigated after treatment of the cells with medium or 250 ng/ml BZB for 16 h. (**B**) Serial dilutions of the molecules were analyzed. (**C**) 20 nM scTRAIL units were analyzed after preincubation of the cells with PBA or a 200-fold molar excess of the respective blocking antibody. Binding of Fc-scTRAIL was measured in the presence of all blocking antibodies separately and is here represented as the mean of blocking with all different antibodies. Bound molecules were detected via anti-FLAG-PE. relative MFI, relative median fluorescence intensity. Pairwise comparisons were performed by unpaired *t* test (two-tailed; ^*^P < 0.05; ^**^P < 0.01; ^***^P < 0.001; ns, *P* > 0.05).

Binding of the fusion proteins to the respective antigens and TRAIL receptors was investigated by ELISA. Half-maximal antigen binding varied from 0.3 nM to 4.3 nM depending on the incorporated antibody moiety with 3- to 14-fold stronger binding of hu225- and 3-43-comprising molecules. In line with previous studies, similar EC_50_ values of 1.0 nM to 1.6 nM were detected for binding to human TRAIL-R2 for all proteins (Supplementary Figure 1, Table 2). Investigations on cross-reactivity confirmed specificity for the designated target antigen for all scFv-Fc-scTRAIL molecules and no binding to EGFR, HER2, HER3, and EpCAM for Fc-scTRAIL (Figure 3A). Furthermore, all scFv-Fc-scTRAIL fusion proteins and non-targeted Fc-scTRAIL bound to all human and mouse TRAIL-receptors, except for moDcTRAIL-R1 (Figure 3B, 3C), which is in accordance with previous studies [37]. Further binding studies were performed by flow cytometry analyzing the colorectal carcinoma cell lines Colo205 and HCT116. First, expression levels of the targeted tumor-associated antigens and TRAIL-receptors were investigated (Figure 4A, Supplementary Table 1). Expression of EGFR was with 23,389 molecules per cell higher on HCT116 than on Colo205 cells (16,721 receptors per cell), while HER2 and HER3 showed considerably higher levels on Colo205 (31,266 HER2; 6,876 HER3) compared to HCT116 cells (6,898 HER2; 2,864 HER3). EpCAM was highly overexpressed on both cell lines (773,023 on Colo205; 423,711 on HCT116). The death receptors TRAIL-R1 and -R2 were detected in higher levels on HCT116 (11,515 TRAIL-R1; 3,859 TRAIL-R2) compared to Colo205 cells (4,291 TRAIL-R1; 1,558 TRAIL-.R2), whereas the decoy receptors TRAIL-R3 and -R4 were expressed in comparably low levels on both cells lines. After treatment of the cells with bortezomib that was used in bioactivity assays to increase sensitivity towards TRAIL-induced apoptosis, Colo205 cells showed significantly upregulated EGFR, TRAIL-R2 and TRAIL-R3 expression. On HCT116 cells, bortezomib treatment significantly increased TRAIL-R2, -R3, and -R4 levels, and induced a significant reduction in EpCAM expression, although EpCAM levels remained high (Figure 4A, Supplementary Table 1). Differing from the ELISA data, the EGFR-targeting fusion protein showed strongest binding to both cell lines, followed by the EpCAM-targeting molecule, the HER2-targeting protein, and the molecules directed against HER3 that did not reach saturating levels in the analyzed concentration range (Figure 4B, Table 2). Of note, even low concentrations of scFv3-43-Fc-scTRAIL displayed stronger binding to Colo205 cells than the non-targeted Fc-scTRAIL, whereas increased binding of scFv3-43-Fc-scTRAIL compared to Fc-scTRAIL was only found for high concentrations on HCT116 cells, indicating an influence of HER3 expression levels (Figure 4B). To further investigate the contribution of the antibody moieties, binding to Colo205 und HCT116 cells was analyzed after preincubation with a 200-fold molar excess of the respective blocking antibody. On Colo205 cells, blocking significantly reduced binding of all targeted molecules, except for scFv3M6-Fc-scTRAIL that showed increased levels under these conditions. On HCT116 cells, only binding of the hu225- and 323/A3hu3-comprising molecules was significantly reduced in the presence of blocking antibody, further supporting a correlation between improved binding by targeting and TAA expression levels. Binding of Fc-scTRAIL remained unaffected by blocking antibodies on both cell lines (Figure 4C).

**Table 2:** Binding properties of scFv-Fc-scTRAIL molecules and Fc-scTRAIL

Protein	Antigen	huTRAIL-R2	Colo205	HCT116
scFvhu225-Fc-scTRAIL	0.9 ± 0.01	1.0 ± 0.02	1.4 ± 1.1	0.4 ± 0.1
scFv4D5-Fc-scTRAIL	4.3 ± 0.6	1.2 ± 0.1	32.5 ± 7.9	(34.8 ± 6.8)^a^
scFv3M6-Fc-scTRAIL	3.3 ± 0.3	1.6 ± 0.2	(>100)^a^	(26.3 ± 0.9)^a^
scFv3-43-Fc-scTRAIL	0.3 ± 0.01	1.2 ± 0.1	(>100)^a^	(>100)^a^
scFv323/A3hu3-Fc-scTRAIL	2.5 ± 0.5	1.4 ± 0.1	7.2 ± 0.4	4.8 ± 0.3
Fc-scTRAIL	n.d.	1.4 ± 0.1	(>100)^a^	(20.9 ± 2.1)^a^

n.d., not determined. ^a^, no saturation reached.

EC_50_ values [nM scTRAIL units] were determined by ELISA (EGFR-, HER2-, HER3-, huTRAIL-R2-Fc, sEpCAM) and flow cytometry (Colo205, HCT116 cells).

### *In vitro* cytotoxicity

Induction of cell death was investigated on Colo205 and HCT116 cells in the absence and presence of the proteasome inhibitor bortezomib (BZB). Bortezomib has been previously described to sensitize various tumor cell lines to TRAIL-induced apoptosis [38]. Thus, bortezomib can stimulate apoptosis by increasing expression of TRAIL receptors and proapoptotic proteins and reducing levels of inhibitors of apoptosis. All molecules induced concentration-dependent cell death on both cell lines in the absence and presence of bortezomib. In the absence of bortezomib, scFvhu225-Fc-scTRAIL and scFv3-43-Fc-scTRAIL showed significantly stronger effects than all other molecules on Colo205 cells (*P <* 0.05), followed by the EpCAM-targeting protein (significantly lower EC_50_ value compared to 4D5, 3M6, and non-targeted constructs with *P <* 0.01), while the remaining targeted molecules exhibited similar or even decreased activity compared to Fc-scTRAIL. In the presence of bortezomib, significantly enhanced cell death induction compared to the non-targeted fusion protein was only found for the hu225- and 3-43-based molecules (*P <* 0.01) (Supplementary Figure 2A, Table 3). HCT116 cells were most efficiently killed by the EGFR- and EpCAM-targeting fusion proteins in absence as well as in presence of bortezomib (*P <* 0.05). All other targeting moieties did not further improve cell death induction compared to non-targeted Fc-scTRAIL (Supplementary Figure 2C, Table 3). Preincubation with the respective blocking antibody reduced the activity of the hu225 and 3-43 molecules on Colo205 cells in the absence and presence of bortezomib (*P <* 0.01) to a level similar to or even less potent as Fc-scTRAIL. However, blocking of EpCAM binding did not change sensitivity towards scFv323/A3hu3-Fc-scTRAIL (Supplementary Figure 2B, Table 3). On HCT116 cells, blocking significantly reduced cell death induction of the EGFR-targeted scTRAIL fusion protein (*P <* 0.01), while the activity of scFv323/A3hu3-Fc-scTRAIL was not influenced to a similar extent (Supplementary Figure 2D, Table 3). With respect to the 18- to 148-fold higher expression levels of EpCAM compared to EGFR, HER2, and HER3 (Figure 4A), the failure to efficiently interfere with the targeting-dependent action of the scTRAIL fusion protein may be related to this high expression level of EpCAM.

**Table 3:** Cell death induction of scFv-Fc-scTRAIL and Fc-scTRAIL fusion proteins

Protein	Colo205			
	without blocking Ab		with blocking Ab	
	without BZB	with BZB	without BZB	with BZB
scFvhu225-Fc-scTRAIL	22.6 ± 3.4	7.9 ± 1.7	106.9 ± 9.0	18.5 ± 3.6
scFv4D5-Fc-scTRAIL	104.4 ± 6.0	29.7 ± 3.5	163.3 ± 9.4	35.6 ± 3.1
scFv3M6-Fc-scTRAIL	247.8 ± 8.8	39.5 ± 5.7	143.8 ± 10.1	26.4 ± 2.9
scFv3-43-Fc-scTRAIL	31.1 ± 3.5	6.8 ± 0.9	217.3 ± 6.5	33.1 ± 5.7
scFv323/A3hu3-Fc-scTRAIL	58.0 ± 12.1	17.7 ± 2.9	31.5 ± 5.7	9.7 ± 1.9
Fc-scTRAIL	96.4 ± 11.5	21.7 ± 3.5	130.0 ± 10.6	35.7 ± 9.7
**Protein**	**HCT116**			
	**without blocking Ab**		**with blocking Ab**	
	**without BZB**	**with BZB**	**without BZB**	**with BZB**
scFvhu225-Fc-scTRAIL	12.9 ± 3.9	1.3 ± 0.2	103.2 ± 22.7	4.0 ± 0.6
scFv4D5-Fc-scTRAIL	109.5 ± 10.7	5.0 ± 1.4	79.2 ± 17.7	4.9 ± 1.6
scFv3M6-Fc-scTRAIL	173.1 ± 27.2	5.7 ± 0.4	125.0 ± 15.3	5.4 ± 1.5
scFv3-43-Fc-scTRAIL	131.1 ± 7.1	3.0 ± 0.8	148.4 ± 31.2	7.1 ± 1.1
scFv323/A3hu3-Fc-scTRAIL	13.5 ± 0.8	1.3 ± 0.3	31.1 ± 6.9	1.0 ± 0.1
Fc-scTRAIL	98.9 ± 22.0	3.2 ± 0.3	75.1 ± 17.3	4.9 ± 0.7

EC_50_ values [pM scTRAIL units] were determined after preincubation of Colo205 and HCT116 cells with medium or 250 ng/ml BZB in the absence or presence of a 200-fold molar excess of the respective blocking antibody (Ab). Fc-scTRAIL was investigated in the presence of a mixture of all blocking antibodies.

**Figure 5:**
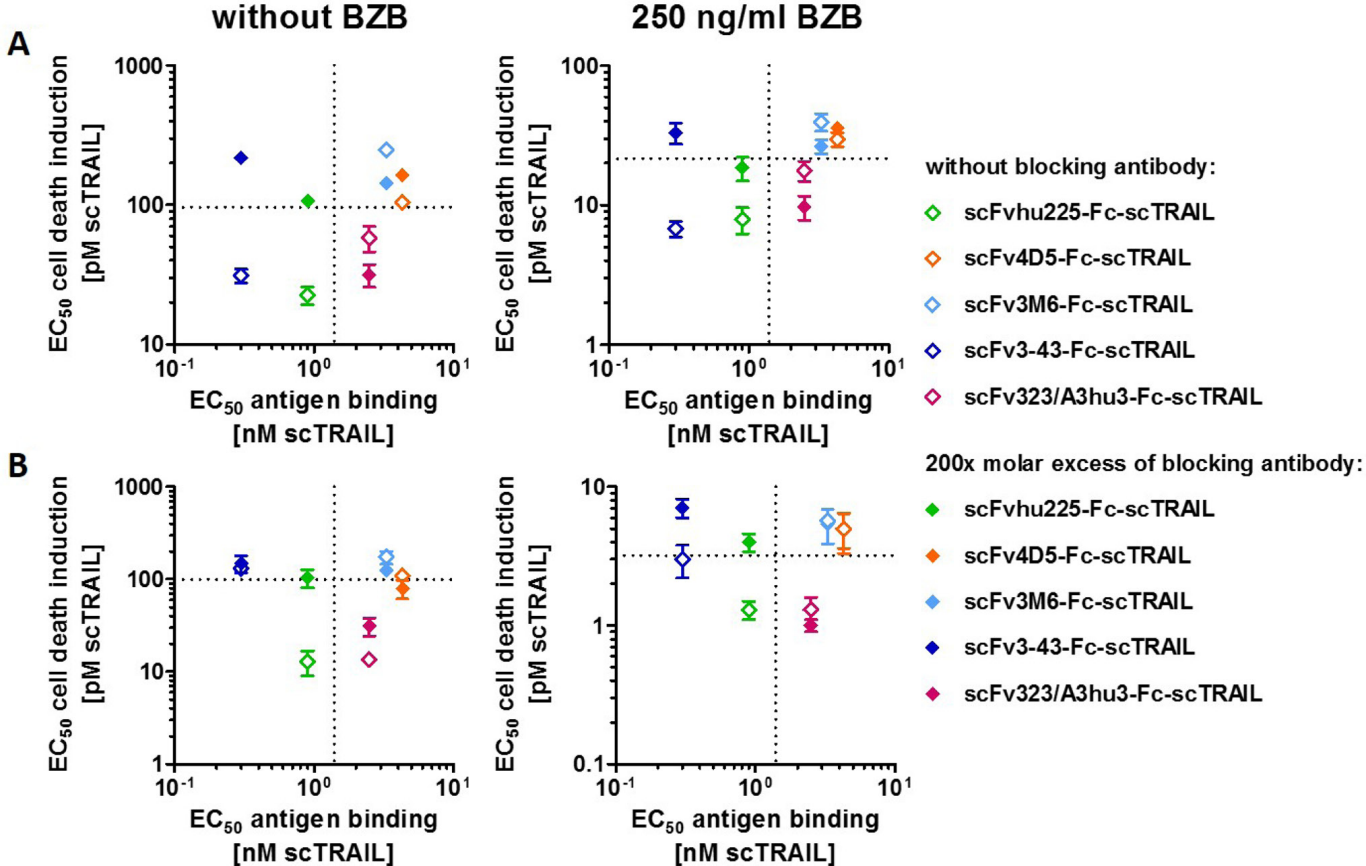
Correlation of cytotoxicity and antigen binding EC_50_ values of cell death induction in the absence and presence of excess amounts of the respective blocking antibody (Table 3) were plotted against the EC_50_ value of binding to the corresponding antigen (Table 2). (**A**) Plots represent data of Colo205 and (**B**) HCT116 cells determined in the absence and presence of 250 ng/ml BZB. EC_50_ values of Fc-scTRAIL for binding to huTRAIL-R2 and cell death induction are indicated with dotted lines.

Investigations of cell death induction revealed that targeting effects were only observed for some of the scFv-Fc-scTRAIL fusion proteins. To unravel potential determinants of *in vitro* targeting effects, EC_50_ values of cell death induction in the absence and presence of blocking antibody were correlated with the EC_50_ values of antigen binding in ELISA. The hu225- and 3-43-comprising molecules that potently induced death of Colo205 cells (that can be reduced to the level of Fc-scTRAIL by blocking antibodies) exhibit strong binding to the corresponding antigen with EC_50_ values lower than those of binding to TRAIL-R2. These relations were observed in the absence and presence of bortezomib (Figure 5A), suggesting that antigen binding by the antibody must be stronger than binding of the scTRAIL moiety to its receptors for *in vitro* targeting effects. However, the results obtained for HCT116 cells do not confirm this correlation (Figure 5B). Consistent with the data of Colo205 cells, scFvhu225-Fc-scTRAIL exerted potent antigen binding and cell death induction that is reduced by cetuximab. In contrast to Colo205 cells, the 3-43 antibody moiety did not improve the activity compared to non-targeted Fc-scTRAIL on HCT116 cells. Considering the binding properties for Colo205 and HCT116 cells (Figure 4B), the hu225 and 3-43 molecules both displayed better binding than Fc-scTRAIL on Colo205 cells, while superior binding to HCT116 cells was only observed for scFvhu225-Fc-scTRAIL, especially at the low concentrations applied for cell death induction. Consistent with these observations, scFv323/A3hu3-Fc-scTRAIL exhibited improved binding and cell death induction compared to Fc-scTRAIL on both cell lines.

### *In vivo* anti-tumor activity

**Figure 6:**
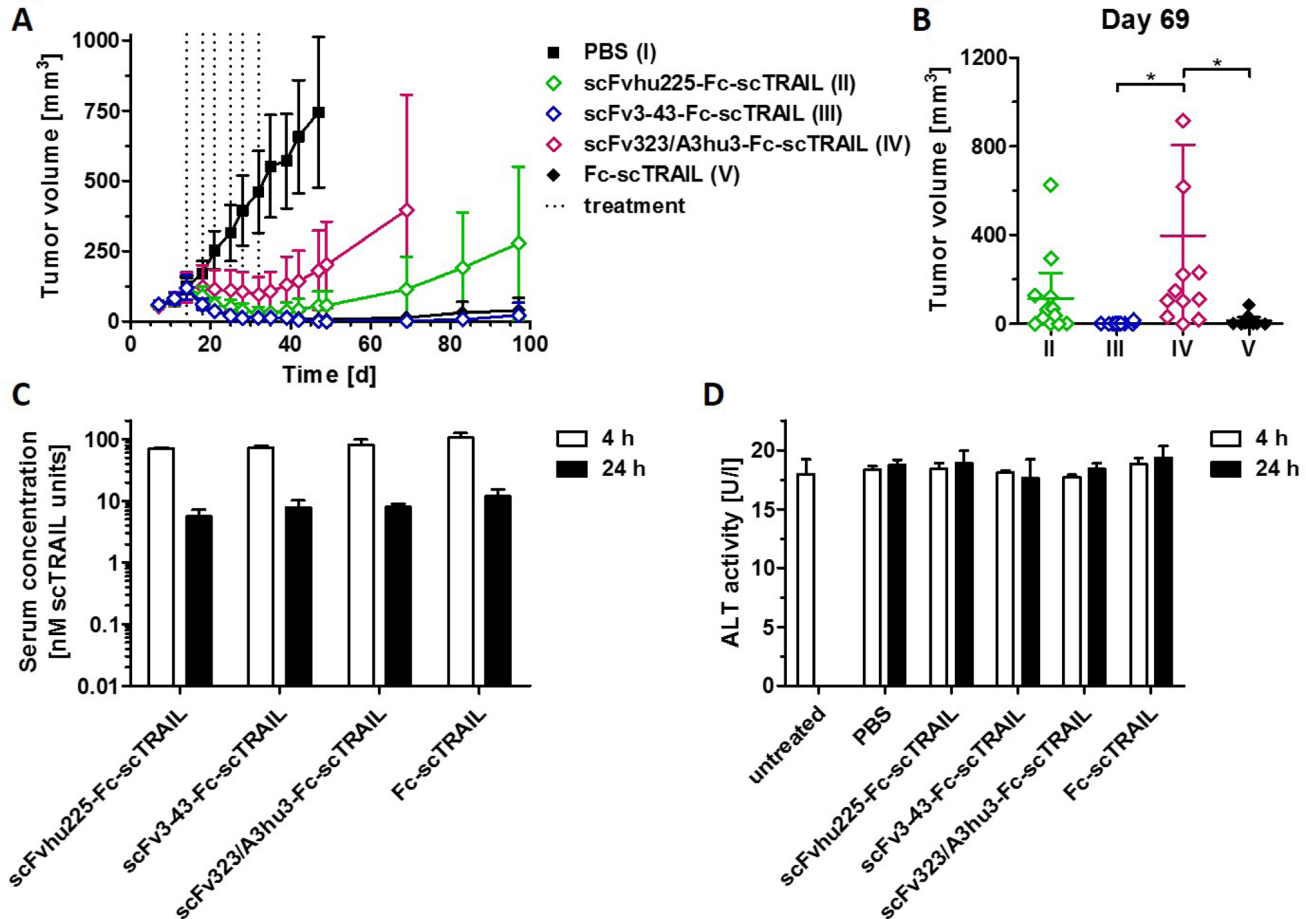
*In vivo* effects of scFv-Fc-scTRAIL molecules and Fc-scTRAIL (**A**) NMRI nude mice (6 mice/group) bearing Colo205 tumors were treated with 0.2 nmol protein (0.4 nmol scTRAIL units; i.v.) or PBS twice a week for three weeks (days 14, 18, 21, 25, 28, 32). Treatments are indicated with dotted lines. (**B**) Statistical analysis of tumor volumes of groups II to V (numbering according to A) at day 69 was performed by One-Way ANOVA, followed by Tukey’s post hoc test (^*^P < 0.05; ^**^P < 0.01; ^***^P < 0.001; ns, *P* > 0.05). (**C**) Serum concentrations of the molecules and D) ALT were determined 4 h and 24 h after the last treatment (day 32).

We compared the *in vivo* anti-tumoral activity of targeted (scFv3-43-Fc-scTRAIL, scFv323/A3hu3-Fc-scTRAIL, scFvhu225-Fc-scTRAIL) and non-targeted Fc-scTRAIL fusion proteins in a subcutaneous Colo205 xenograft model, injecting 0.2 nmol protein (corresponding to 0.4 nmol scTRAIL units) twice a week for three weeks. The EGFR-, HER3-, and non-targeted molecules induced almost complete tumor remission, whereas only partial tumor regression and fast regrowth were found for scFv323/A3hu3-Fc-scTRAIL (Figure 6A). Treatment with scFv3-43-Fc-scTRAIL resulted in stable tumor remission over the monitoring period of almost 100 d and only marginal regrowth was observed for Fc-scTRAIL at the end of the experiment. In contrast, tumors treated with scFvhu225-Fc-scTRAIL showed earlier regrowth. Statistical analysis revealed significantly higher tumor volumes of scFv323/A3hu3-Fc-scTRAIL- compared to scFv3-43-Fc-scTRAIL- and Fc-scTRAIL-treated mice at day 69 (Figure 6B). Serum concentrations of the proteins determined 4 h and 24 h after the last treatment were similar for all molecules (Figure 6C). Thus, incorporation of different targeting moieties did not substantially alter clearance. Safety of all treatments was confirmed by measuring ALT levels in the serum 4 h and 24 h after the last treatment (Figure 6D).

## DISCUSSION

In previous studies, we showed that dimeric assembly of single-chain versions of TRAIL, enabling hexavalent TRAIL receptor interactions, overcomes the limited efficacy of monomeric, trivalent scTRAIL. Combining these dimeric scTRAIL fusion proteins with an EGFR-targeting antibody moiety even further improved their activity [19–21, 28]. Fusion to an Fc region as dimerization module has emerged as promising strategy, due to an extended half-life of the respective fusion proteins, although TRAIL-mediated clearance effects result in rather short half-lives in mice of around 15 to 28 hours [27, 28]. Compared to diabody-scTRAIL and EHD2-scTRAIL fusion proteins, exhibiting even shorter half-lives, a superior antitumor activity was observed for Fc-scTRAIL and EGFR-targeting scFv-Fc-scTRAIL fusion proteins [28]. Here, we further investigated the effects of targeting to tumor-associated antigens employing five scFvs of distinct specificity and affinity for construction of targeted, Fc-comprising scTRAIL fusion proteins and a side-by-side comparison of their functional activity *in vitro* and *in vivo*. Conceptually, active targeting through fusion with an antibody moiety should i) result in increased accumulation of the fusion proteins at the target site, i.e. increase the local concentration in the tumor, and ii) result in a membrane-like presentation of TRAIL, i.e. generating a multivalent TRAIL surface leading to receptor clustering and efficient activation of cell death signals [8, 39].

The finding that only some of the scFv-Fc-scTRAIL molecules showed superior *in vitro* activity compared to non-targeted Fc-scTRAIL was unexpected based on our previous results with EGFR-targeting scTRAIL fusion proteins [19]. To elucidate potential underlying factors that influence targeting effects, we correlated EC_50_ values of cell death induction with the binding properties of the respective molecule. Two important parameters emerged from this analysis. First, the affinity of the antibody moiety for its antigen should be higher than that of the TRAIL - TRAIL receptor interaction to result in a positive effect of tumor targeting on TRAIL cell death induction. Second, evident from different HER3 expression levels of Colo205 and HCT116 cells, respectively, antigen density might also affect efficacy of targeting. Accordingly, targeting effects more likely become apparent on tumor cells overexpressing the tumor-associated marker. However, as deduced from data for the EpCAM-targeting scFv-Fc-scTRAIL fusion protein, high levels of expressed target antigen do not necessarily result in most potent targeting effects. An increased cytotoxicity through targeting requires a simultaneous binding of the antibody-TRAIL fusion proteins to the target antigen and TRAIL receptors, i.e. a spatial co-localization either on the same or neighboring cells [16], which might be less efficient for the fusion protein directed against EpCAM.

The data reported here contrast our and other’s previous findings with monomeric scFv-(sc)TRAIL fusion proteins, where a strict target dependency on *in vitro* and *in vivo* efficacy was noted [8, 18], despite K_D_ values of some antibody moieties were as high as 142 nM [40]. In the absence of target antigen expression, these monomeric scFv-(sc)TRAIL fusion proteins, exert a considerably lower bioactivity compared to the novel dimeric, i.e. hexavalent formats of scTRAIL described here, which are intrinsically capable of activating TRAIL-R2, whereas efficient TRAIL-R2 activation by the monomeric scTRAIL fusion proteins requires target antigen binding to facilitate death receptor clustering and activation. Thus, mechanistically there is a fundamental difference between trivalent and hexavalent TRAIL fusion proteins. Trivalent TRAIL fusion proteins require binding to a cell membrane, which can be achieved by fusion to an antibody moiety, to induce efficient clustering and activation of TRAIL receptor 2, while hexavalent TRAIL, even without targeting, induces efficient activation of both death receptors. Anti-tumor effects might also be influenced by the size of the fusion protein, affecting vascular permeability as well as tumor penetration and retention [41]. Here, smaller molecules, such as the Fc-scTRAIL, might have advantages over the scFv-Fc-scTRAIL fusion proteins. Furthermore, high affinity binding to TAA-expressing tumor cells of the targeted fusion proteins might restrict penetration of the targeted fusion protein into the tumor tissue by a binding-site barrier [42].

Besides actively directing the scTRAIL fusion proteins to tumor cells, the investigated antibody moieties have the potential to block proliferative signaling of the targeted receptors. Due to activating mutations in the MAPK and PI3K pathways, EGFR-, HER2-, and HER3-antagonistic antibodies are not able to block downstream signaling in the Colo205 and HCT116 cells used here. However, blocking EGFR signaling by a scTRAIL molecule comprising the hu225 antibody has been demonstrated in wild-type KRAS-expressing cells [43], highlighting the possible multiple modes of action of antibody-scTRAIL fusion proteins.

Additionally, indirect TRAIL-induced pro-survival signaling via EGFR/HER2 has been reported in colorectal cancer cells [44], which provides further rationale for combination of TRAIL with EGFR- and HER2-blocking molecules. Indeed, combination of an anti-HER2 and an anti-TRAIL-R2 antibody showed synergistic effects in a mouse model of breast cancer [45]. Alternatively, EpCAM-blocking antibodies provide a means of selectively killing cancer stem cells and inhibiting EpCAM’s mitogenic signaling activity [46]. In the present study, EpCAM-targeting scFv323/A3hu3-Fc-scTRAIL showed significantly improved cell death induction compared to Fc-scTRAIL *in vitro*. Our data show that careful selection of an antibody moiety with respect to binding strength, specificity and interference with the signaling pathway of the targeted receptor has the potential to enhance the *in vitro* bioactivity of dimeric scTRAIL fusion proteins. In line with this, an appropriate targeting moiety according to properties of the individual tumor appears necessary to achieve an optimum *in vivo* tumor response. Furthermore, tumor targeting may help to restrict the action of the applied protein therapeutic to the tumor, thereby increasing the therapeutic window. This reasoning is supported by recent studies comparing EGFR-targeted and non-targeted scTRAIL on HCC, healthy human liver cells and intact liver tissue, revealing superior tolerability of the targeted molecule [47].

In conclusion, our data highlight the complexity of targeting hexavalent TRAIL molecules to tumor cells for increased cytotoxicity, but also show that by careful selection of a tumor-targeting antibody it should be possible to further improve the anti-tumoral activity of *a priori* powerful hexavalent Fc-scTRAIL molecules in order to achieve lasting, complete tumor remissions.

## MATERIALS AND METHODS

### Materials

Receptor-Fc and scFv-Fc fusion proteins were produced with stably transfected HEK293T cells and purified from the supernatant by protein A affinity chromatography as described previously [19, 30]. Mouse TRAIL-R2-Fc was purchased from Sino Biological Inc. (50412-M03H). HRP- and PE-conjugated anti-FLAG antibodies were obtained from Sigma-Aldrich (A8592) and Miltenyi Biotec (130-101-576), respectively. For *in vitro* studies, bortezomib (BZB) was purchased from UBPBio (F1200). Clinical grade bortezomib, cetuximab, and trastuzumab were kindly provided by Dr. Thomas Mürdter and Dr. Jens Schmid (Dr. Margarete Fischer-Bosch-Institute of Clinical Pharmacology, Stuttgart, Germany). The human colon carcinoma cell lines Colo205 and HCT116 were obtained from ATCC in 2012 without further authentication and tested negative for Mycoplasma (MycoAlert, Lonza) before making master stocks. Cells were cultured in RPMI 1640 (Thermo Fisher Scientific, 11875), supplemented with 10% (v/v) FBS (PAN Biotech, 3302-P121707) for a maximum of 2 months. For animal experiments, cells were cultured for 2–3 weeks from freshly thawed master stocks. Anti-FLAG^®^ M2 affinity gel was obtained from Sigma-Aldrich (A2220). FLAG peptide was purchased from peptides&elephants (EP01741). BD OptEIA™ human TRAIL ELISA set and Alanine Transaminase Assay kit were obtained from BD Biosciences (550948) and abcam (ab105134), respectively. NMRI nude mice were purchased from Charles River.

### Cloning and production of scTRAIL fusion proteins

Modular vectors for sequential insertion of the Fc region, scTRAIL, and the antibody moiety were created based on pSecTagAL1, a modified version of pSecTagA (Invitrogen, Thermo Fisher Scientific, V90020) comprising an AgeI restriction site at the 3’-end of the Igκ chain leader sequence. In order to eliminate potential protease cleavage sites, K447 of the human IgG1 Fc (EU numbering scheme) was mutated to Q. Single-chain variable fragments hu225 [30], 4D5 [31], 3M6 (derived from MM-121, Ab #6 [32] comprising a cysteine to serine mutation at position 89 (Kabat numbering scheme) in the variable domain of the light chain), 3–43 [33], and 323/A3hu3 [34] directed against EGFR, HER2, HER3, and EpCAM were used. A single-chain version of TRAIL (scTRAIL) containing amino acid residues 118–281 of human TRAIL and a linker composed of a single glycine residue was used to create the various fusion proteins [21]. All scTRAIL fusion proteins were produced in stably transfected HEK293T cells (cultivated in Opti-MEM (Thermo Fisher Scientific, 31985–070) supplemented with 50 µM ZnCl_2_) and purified from the supernatant via FLAG affinity chromatography according to the manufacturer’s protocol. Proteins were eluted with 100 µg/ml FLAG peptide and dialyzed against PBS. Optionally, size-exclusion FPLC was performed as additional purification step applying concentrated protein on a Superdex 200 10/300 GL column (PBS as mobile phase, 0.5 ml/min flow rate). The protein concentration was measured with a spectrophotometer (NanoDrop ND-1000) using the calculated molecular mass and molar extinction coefficient. Aliquots were stored at –80° C.

### Biochemical characterization of scTRAIL fusion proteins

Proteins were evaluated by SDS-PAGE under reducing and non-reducing conditions and stained with Coomassie Brilliant Blue G-250. HPLC size-exclusion chromatography (SEC) was performed using a Phenomenex Yarra 3 µm SEC-2000 or -3000 column (Phenomenex, 00H-4512-K0 or 00H-4513-K0), a Waters 2695 HPLC, and 0.1 M Na_2_HPO_4_/NaH_2_PO_4_, 0.1 M Na_2_SO_4_, pH 6.7 as mobile phase (0.5 ml/min flow rate). Thyroglobulin (669 kDa, S_*r*_ 8.5 nm), β-amylase (200 kDa, S_*r*_ 5.4 nM), bovine serum albumin (67 kDa, S_*r*_ 3.55 nm), carbonic anhydrase (29 kDa, S_*r*_ 2.35 nm), and FLAG peptide (1 kDa) were used as reference proteins. The thermal stability was analyzed by dynamic light scattering using a ZetaSizer Nano ZS (Malvern) using 100 µg protein diluted to a volume of 1 ml in PBS. Mean count rates were measured increasing the temperature from 35° C to 90° C in 1° C intervals and equilibrating the sample for 2 min at each step. The melting point was defined as the temperature showing an increase in the mean count rate.

### ELISA

EGFR-, HER2-, HER3-, TRAIL-R-Fc fusion proteins or sEpCAM were coated overnight at 4° C and remaining binding sites were blocked with 2% (w/v) non-fat dry milk/PBS (MPBS). Serial dilutions or single concentrations of the purified proteins in MPBS were analyzed in duplicates. Bound molecules were detected with anti-FLAG-HRP or anti-human IgG (Fc specific)-peroxidase (Sigma-Aldrich, A0170) using 100 µl of 3,3’,5,5’-tetramethylbenzidine (TMB) substrate per well (0.1 mg/ml TMB, 100 mM sodium acetate buffer, pH 6.0, 0.006% (v/v) H_2_O_2_). Reaction was stopped with 1 M H_2_SO_4_ (50 µl/well), and optical density was measured at 450 nm in an ELISA reader.

### Flow cytometry

Absolute receptor expression levels were determined using QIFIKIT^®^ (Dako, K007811-8) according to the manufacturer’s protocol and the following antibodies: anti-EGFR (BioLegend, 352902), anti-HER2 (BioLegend, 324402), anti-HER3 (BioLegend, 324702), anti-EpCAM (BioLegend, 324202), anti-TRAIL-R1 (R&D Systems, MAB347), anti-TRAIL-R2 (R&D Systems, MAB6311), anti-TRAIL-R3 (R&D Systems, MAB6301), anti-TRAIL-R4 (R&D Systems, MAB633) antibodies and moIgG1κ (BioLegend, 4001202), moIgG2aκ (BioLegend, 400202), moIgG2bκ (BioLegend, 401202) isotype controls. Prior to detection of receptor molecules, cells were cultivated in medium or medium containing 250 ng/ml BZB for 16 h. To analyze binding of the scTRAIL fusion proteins to Colo205 and HCT116 cells, 250,000 cells/well were seeded and incubated with serial dilutions of the proteins in PBS, 2% (v/v) FBS, 0.02% (w/v) sodium azide (PBA) for 1 h at 4° C. Bound molecules were detected with an anti-FLAG-PE antibody. Binding to EGFR, HER2, HER3, or EpCAM was blocked by preincubation of the cells with a 200-fold molar excess of the respective blocking antibody (IgG or scFv-Fc) for 30 min, prior to addition of the scTRAIL fusion proteins to a final concentration of 20 nM (scTRAIL units). Incubation and detection steps were done as described above. Determination of median fluorescence intensities was performed using FlowJo. Relative median fluorescence intensities were calculated using the equation relative MFI = (MFI_sample_-(MFI_detection system_-MFI_cells_))/MFI_cells_.

### *In vitro* cytotoxicity

Colo205 (50,000/well) or HCT116 (15,000/well) cells were seeded in 100 µl medium and cultivated at 37° C, 5% CO_2_ overnight. Prior to treatment with the scTRAIL fusion proteins for 16 h, cells were preincubated with bortezomib (final concentration 250 ng/ml) or medium for 30 min. Killing controls were treated with 0.25% (v/v) Triton X100. Viable cell were stained with crystal violet. Staining was solved in methanol (50 µl/well), and optical density was measured at 550 nm. To analyze the effects of targeting on cell death induction, a 200-fold molar excess of the respective blocking antibody (IgG or scFv-Fc) was added during the preincubation step described above.

### Pharmacokinetics and pharmacodynamics

Animal care and all experiments performed were in accordance with federal and European guidelines and have been approved by university and state authorities. A Colo205 xenograft model was used to analyze the anti-tumoral activity *in vivo*. 3 × 10^6^ Colo205 cells (in 100 µl PBS) were injected subcutaneously into the left and right flank of female NMRI nude mice (9 and 11 weeks old, 6 mice per group). Tumor size was monitored by measuring the length (a) and width (b) of the tumors with a caliper, and calculation of the tumor volume by the equation a x b^2^/2. Upon reaching a volume of ∼100 mm^3^, treatment was started. Mice received i.v. injections of 0.2 nmol protein (0.4 nmol scTRAIL units, in 150 µl PBS) or PBS as control twice a week (every fourth and third day, respectively) for three weeks. Blood samples were collected 4 h and 24 h after the last treatment, incubated on ice for 30 min, and centrifuged at 13,000 g for 30 min at 4° C. Serum concentrations of scTRAIL molecules (serum samples stored at 20° C were determined using BD OptEIA™ human TRAIL ELISA set according to the manufacturer’s protocol. Serum concentrations of the proteins were obtained by interpolation from a standard curve of the purified protein.

### ALT assay

Blood samples taken 4 h and 24 h after the last treatment of the pharmacodynamic experiment were analyzed for potential induction of liver toxicity. Serum was stored at 4° C, and serum levels of alanine transaminase were determined using Alanine Transaminase Assay kit (abcam) according to the manufacturer’s instructions. 10 µl of serum samples were analyzed.

### Statistics

Except for tumor volumes that are expressed as mean ± 95% CI, all data are represented as mean ± SD of at least three independent experiments. Pairwise and multiple comparisons were performed by unpaired *t* test (two-tailed) and One-Way ANOVA, followed by Tukey’s post hoc test, respectively (GraphPad Prism, GraphPad Software). A *P* value of < 0.05 was considered statistically significant (^*^P < 0.05; ^**^P < 0.01; ^***^P < 0.001; ns, *P* > 0.05). Block shift was only performed to compensate differences in OD values due to varying ELISA developing times and is clearly indicated. The corrected value of the experiment n (X_n_’) was calculated from the measured value of experiment n (X_n_), the duplicate average of experiment n (Y_n_), and the average of all measured values of all experiments (Ȳ) using the equation X_n_’ = X_n_ – (Y_n_–Ȳ).

## SUPPLEMENTARY MATERIALS FIGURES AND TABLE



## Abbreviations

ALT: alanine transaminase
BZB: bortezomib
DR: death receptor
EGFR: epidermal growth factor receptor
EHD2: IgE heavy chain domain 2
EpCAM: epithelial cell adhesion molecule
Fc: fragment crystallizable
HER2/3: human epidermal growth factor receptor 2/3
PARA: pro-apoptotic receptor agonists
scFv: single-chain Fragment variable
scTRAIL: single-chain tumor necrosis factor-related apoptosis-inducing ligand
SEC: size exclusion chromatography
sTRAIL: soluble tumor necrosis factor-related apoptosis-inducing ligand
TAA: tumor-associated antigen
TNF: tumor necrosis factor
TRAIL: TNF-related apoptosis-inducing ligand
