# Supplementary material for: Targeting scFv-Fc-scTRAIL fusion proteins to tumor cells
Source: Oncotarget. 2018 Jan 31;9(13):11322–35. doi: 10.18632/oncotarget.24379 (PMC5834252; doi:10.18632/oncotarget.24379)
Supplement: Supplementary file 1 [file oncotarget-09-11322-s001.pdf]

Targeting scFv-Fc-scTRAIL fusion proteins to tumor cells

SUPPLEMENTARY MATERIALS

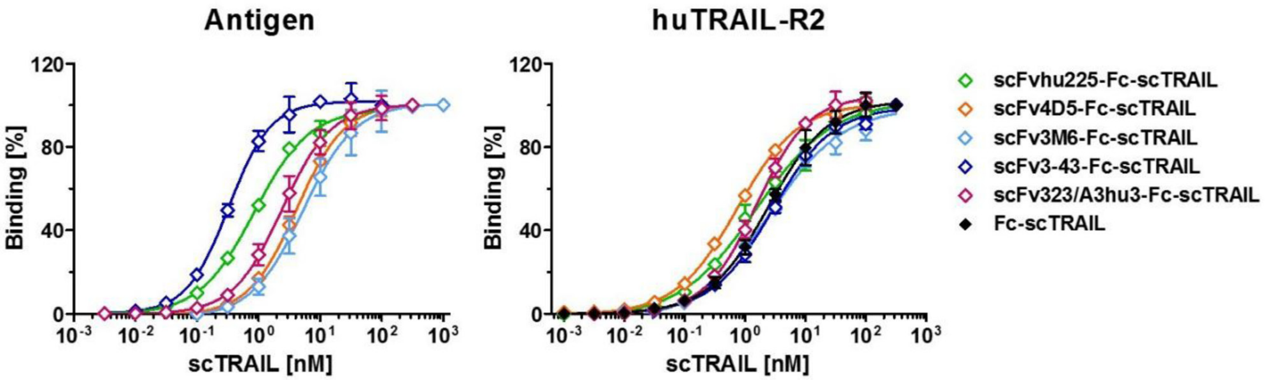

Supplementary Figure 1: Binding of targeted and non-targeted molecules to the respective antigen and human TRAIL-R2 in ELISA. EGFR-Fc, HER2-Fc (each 300 ng/well), HER3-Fc, sEpCAM, or huTRAIL-R2-Fc (each 200 ng/well) were coated and bound molecules detected via anti-FLAG-HRP or anti-human IgG (Fc specific)-POD.

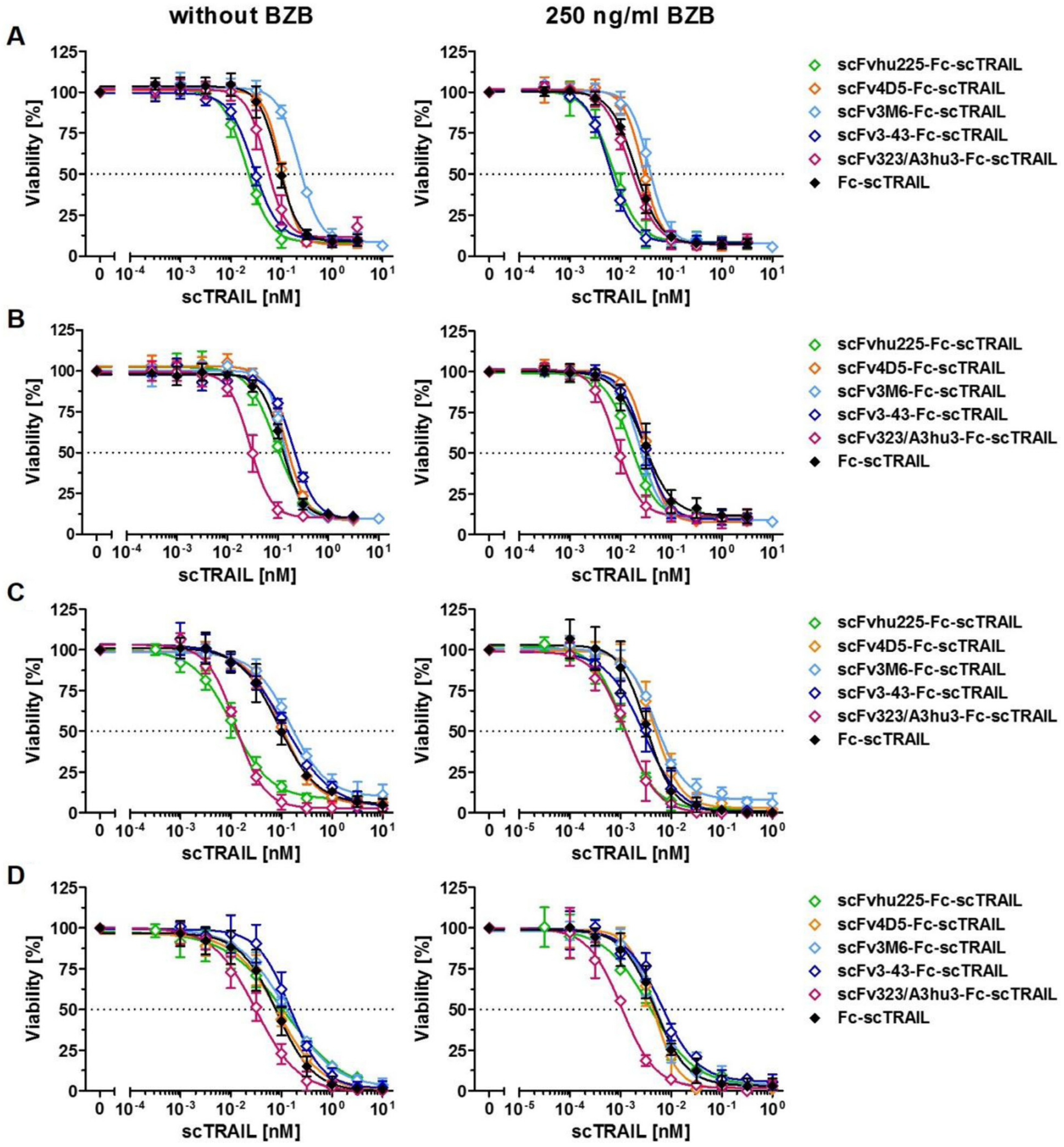

Supplementary Figure 2: Cell death induction of scFv-Fc-scTRAIL molecules and Fc-scTRAIL. (**A**) Colo205 cells were analyzed after preincubation with medium or 250 ng/ml BZB in the absence and (**B**) presence of a 200-fold molar excess of the respective blocking antibody. Fc-scTRAIL was investigated in the presence of a mixture of all blocking antibodies. (**C**) Killing of HCT116 cells was investigated after preincubation with medium or 250 ng/ml BZB in the absence and (**D**) presence of a 200-fold molar excess of the respective blocking antibody. Fc-scTRAIL was investigated in the presence of a mixture of all blocking antibodies.

Supplementary Table 1: Receptor expression levels of Colo205 and HCT116

Receptor	Colo205	Colo205 + BZB	HCT116	HCT116 + BZB
EGFR	16,721 ± 751	18,979 ± 461	23,389 ± 1,506	19,307 ± 2,298
HER2	31,266 ± 4,787	30,793 ± 4,156	6,898 ± 886	10,064 ± 4,935
HER3	6,876 ± 1,114	6,552 ± 613	2,864 ± 1,027	4,959 ± 2,249
EpCAM	773,023 ± 45,950	919,793 ± 83,669	423,711 ± 23,024	371,850 ± 10,123
TRAIL-R1	4,291 ± 912	8,137 ± 2,199	11,515 ± 4,197	9,865 ± 3,382
TRAIL-R2	1,558 ± 280	4,879 ± 1,056	3,859 ± 2,251	14,293 ± 617
TRAIL-R3	1,372 ± 500	2,821 ± 264	1,964 ± 486	6,058 ± 1,304
TRAIL-R4	1,617 ± 862	1,535 ± 396	1,476 ± 215	4,995 ± 1,052

Receptor expression levels of cells cultivated in medium or medium containing 250 ng/ml BZB were determined by flow cytometry.
